# Three-Dimensional Gait Analysis in Children Undergoing Gastrocsoleus Lengthening for Equinus Secondary to Cerebral Palsy

**DOI:** 10.3390/medicina57020098

**Published:** 2021-01-22

**Authors:** Norine Ma, Nicholas Sclavos, Elyse Passmore, Pam Thomason, Kerr Graham, Erich Rutz

**Affiliations:** 1 Australia and Hugh Williamson Gait Laboratory, Pediatric Orthopedic Department, The Royal Children’s Hospital, Parkville, Melbourne, VIC 3052, Australia; norinem@student.unimelb.edu.au (N.M.); nsclavos@student.unimelb.edu.au (N.S.); Elyse.Passmore@rch.org.au (E.P.); Pam.Thomason@rch.org.au (P.T.); Kerr.Graham@rch.org.au (K.G.); 2 Murdoch Children’s Research Institute, Melbourne, VIC 3052, Australia; 3 Faculty of Engineering and Information Technology, The University of Melbourne, Melbourne, VIC 3052, Australia; 4 Department of Pediatrics, The University of Melbourne, Melbourne, VIC 3052, Australia; 5 Medical Faculty, The University of Basel, 4001 Basel, Switzerland

**Keywords:** cerebral palsy, equinus, gastrocsoleus lengthening, gait analysis

## Abstract

*Background and Objectives:* Equinus is the most common deformity in children with cerebral palsy, and surgical lengthening of the gastrocsoleus muscle-tendon unit is the most commonly performed operation for children with cerebral palsy. Treatment outcomes of orthopaedic surgery can be measured objectively with three-dimensional gait analysis. This study examined the quality of evidence for gastrocsoleus lengthening surgery based on objective measures. *Materials and Methods:* A search was performed with Medline, Embase and PubMed from 1990 to 25 August 2020 using the keywords “cerebral palsy”, “equinus”, “surgery” and “gait analysis”. Only studies of gastrocsoleus lengthening surgery using three-dimensional gait analysis were included, yielding 34 studies. *Results:* Fourteen studies reported swing phase kinematics and all studies reported a significant improvement. Rates of recurrent equinus and calcaneus were reported in 21 studies and varied widely based on follow-up period and surgical technique. *Conclusions:* Poor study quality and marked variability in study samples and interventions made comparison difficult. Future studies should consider prospective design, controls or comparison groups and more detailed breakdowns of outcomes by cerebral palsy subtype, sagittal gait pattern, and equinus type in order to allow more rigorous treatment recommendations to be made.

## 1. Introduction

Cerebral palsy (CP) is caused by non-progressive brain injury during early development [1]. Equinus is the most common gait abnormality in children with CP and is characterised by excessive ankle plantarflexion during stance phase [2]. This is often accompanied by persistent plantarflexion during swing phase, commonly referred to as ‘drop foot’. Equinus deformity is not only caused by contracture of the gastrocsoleus muscle, but can also involve abnormal activity of other muscles of the ankle joint, such as tibialis anterior and peroneus longus [3,4,5]. Dynamic equinus may be treated non-operatively with methods including physiotherapy, injections of Botulinum Neurotoxin A (BoNTA), serial casting or ankle-foot orthoses (AFOs). Over time, dynamic equinus usually progresses to fixed contracture, which is treated surgically by lengthening of the gastrocsoleus muscle-tendon unit (MTU) by a variety of techniques [6].

Clinical examination, observational gait analysis (OGA) and video recording of gait (VGA) can be utilised to measure treatment outcomes. These measures are largely subjective, with inherent problems of validity and reliability [7]. This makes interpretation of studies difficult, and synthesis of multiple studies impossible. Three-dimensional gait analysis (3DGA) is considered the “gold standard” for assessing gait and measuring outcomes. It is objective, reliable and allows detailed analysis of gait parameters. Global gait scores transform multiple kinematic variables from 3DGA into single objective measures of the deviation of a patient’s overall gait pattern from typically developing controls (TDC) [8]. This is useful in defining the cumulative effects of either single level surgery or single-event multi-level surgery (SEMLS).

Surgical treatment for equinus has been shown to improve ankle kinematics during stance phase in children with both unilateral spastic cerebral palsy (USCP) and bilateral spastic cerebral palsy (BSCP) [9,10,11]. However, deviations from typically developing children’s gait patterns may persist during swing phase, with many patients expressing dissatisfaction because of persistent drop foot, which is appreciated as a limp [12]. Understanding pre-operative predictors for post-operative outcomes is crucial to improve treatment planning.

**Figure 1:**
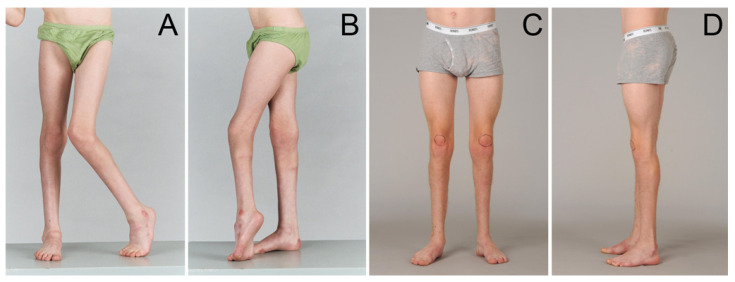
Surgical correction of equinus in a 10 year old boy with asymmetric BSCP (GMFCS II) before (**A**,**B**) and after (**C**,**D**) Single Event Multi-level Surgery (SEMLS), which included surgery for equinus deformity as well as for proximal deformities. The postoperative images were at 5-year follow-up and show effective and permanent correction of equinus deformity and equinus gait. SEMLS surgery was planned (**A**,**B**) using 3DGA and the outcome (**C**,**D**) was assessed using 3DGA. Copyright Prof. Kerr Graham, The Royal Children’s Hospital, Melbourne, Australia. Illustration reproduced with permission from Prof. Graham.

**Figure 2:**
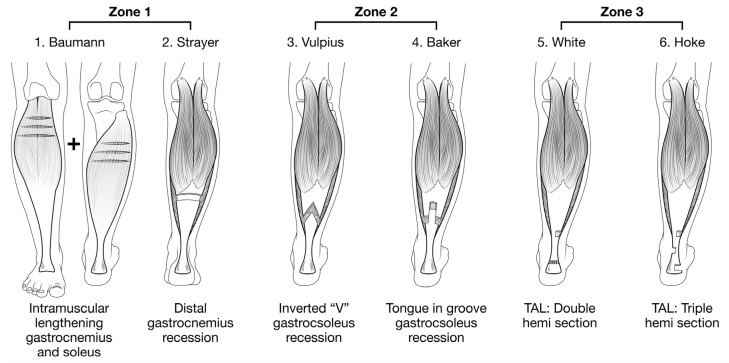
Surgical techniques for gastrocsoleus lengthening (GSL) based on the Zonal classification of the gastrocsoleus muscle-tendon unit. Six of the more than 12 techniques for GSL are illustrated, with two eponymous techniques shown in each Zone. See Reference [1] for a more detailed discussion. Illustration reproduced by permission. Copyright Prof. Kerr Graham, The Royal Children’s Hospital, Melbourne, Australia. Illustration reproduced with permission from Prof. Graham.

This review provides an overview of the current evidence for surgical correction of equinus deformities in CP based on the use of 3DGA for both planning and assessing outcomes (please see Figure 1 and Figure 2). It focuses on the outcomes of surgical management measurable using 3DGA, particularly swing phase dorsiflexion to investigate drop foot, gait scores to understand impact on overall gait pattern and rates of recurrent equinus and calcaneus to consider stability of correction.

## 2. Materials and Methods

A search using keywords was performed with Medline and Embase using Ovid, with a date range from 1990 to 25 August 2020. PubMed was searched using keywords only to retrieve E-pubs and items not indexed in Medline. The Medline search strategy was adapted for use in other databases; the search histories are listed in the Supplementary Information. One additional study was obtained by direct contact with the author. Results were limited to English language only.

Duplicates were removed and titles and abstracts were screened. Studies with fewer than 10 participants were excluded, as well as diagnoses other than CP, and studies without original data. Studies of children aged zero to 18 were included. Full text articles were then screened to include studies using 3DGA. The original search strategy was used to find studies covering both non-operative and operative treatment options. However, for the purposes of this review, only studies of calf lengthening surgery were included. Sample demographics, interventions, and outcome measures for ankle kinematics were extracted from included studies.

Cohort studies were evaluated for methodological quality by the first and senior author using the Methodological Index for Non-Randomized Studies (MINORS) tool. MINORS is a valid instrument for assessing the quality of non-randomised surgical studies, based on criteria such as methods of patient recruitment, data collection, presence of a control group or comparison group, and the duration and completeness of follow-up [13].

## 3. Results

**Figure 3:**
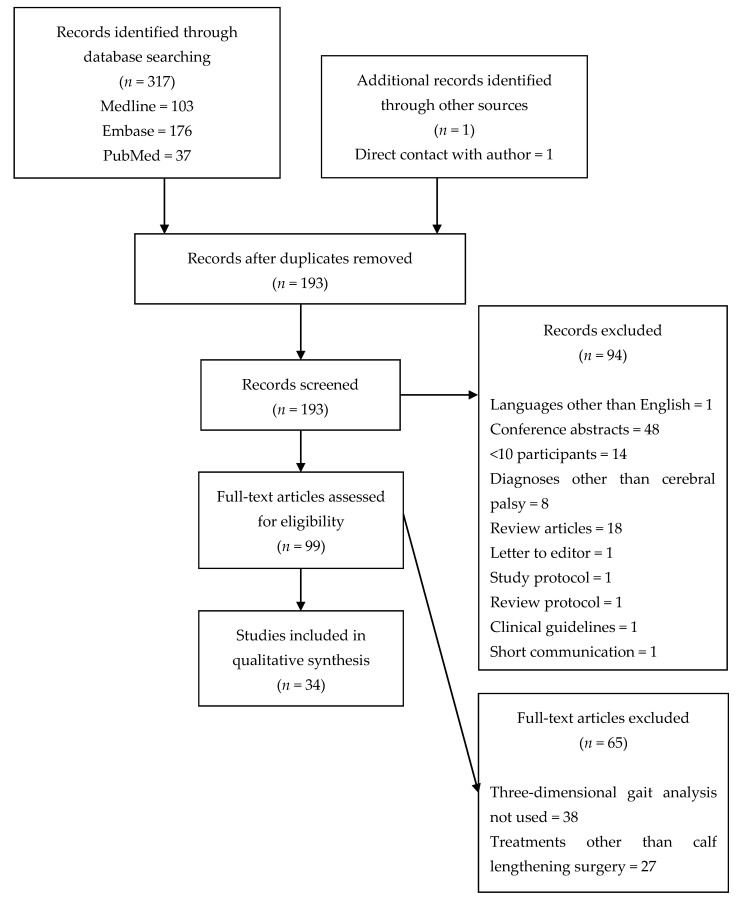
PRISMA diagram.

The search identified 103 articles in Medline, 176 articles in Embase, 37 articles in PubMed, and one article from direct contact with the author. After removal of duplicates, 193 articles remained, of which 34 met the inclusion criteria. Exclusion criteria and number of studies in each step of the search are described in a PRISMA diagram (Figure 3).

Thirty-three cohort studies and one Delphi consensus study of calf lengthening surgery for equinus were identified (Table 1). These included isolated calf lengthening and SEMLS. Follow-up ranged from three to 156 months. Sample sizes ranged from 10 to 134 patients. Definitions of equinus were reported in thirteen studies [10,11,12,14,15,16,17,18,19,20,21,22,23] with various thresholds of sagittal ankle kinematics used. Eleven studies [9,11,14,16,20,21,22,23,24,25,26] specified the inclusion of fixed equinus, but not all used the widely used Silfverskiold test. Seventeen [9,11,12,14,16,18,19,20,24,27,28,29,30,31,32,33,34] studies specified the CP subtype as spastic CP.

### 3.1. Swing Phase Dorsiflexion

Fourteen studies reported swing phase kinematics [4,9,10,12,14,18,21,23,24,25,26,33,35,36]. All studies reported significant increases in maximum or mean swing phase ankle dorsiflexion. However, effect size and power were not reported in any study. Improvement in maximum dorsiflexion in swing in patients with BSCP ranged from 8.0° to 17.7°, whereas improvement in USCP ranged from 12.0° to 20.9°. These improvements are clinically meaningful but the Minimal Clinically Important Difference (MCID) has only been established for composite gait indices, such as the Gait Profile Score (GPS).

### 3.2. Gait Scores

Studies using gait scores are shown in Table 2.

Four studies reported Gait Deviation Index (GDI) [11,29,32,37]. GDI combines 15 gait features to measure overall gait pathology. An improvement in 10 points of GDI corresponds to one standard deviation towards the mean for typically developing children [38]. Improvement in GDI ranged from 12.5 to 24.3 in patients with BSCP. Improvement in GDI of 10.6 for USCP was reported by one study [32].

Three studies reported Gillette Gait Index (GGI) [9,32,39]. GGI combines 16 kinematic parameters to measure overall gait, with higher scores indicating greater deviation from able-bodied individuals [40]. Improvement in mean GGI for USCP ranged from 157 to 200, while improvement in BSCP ranged from 184 to 337.

Seven studies [16,19,20,23,30,32,33] reported ankle Gait Variable Score (GVS). GVS measures the difference between patients and people with no gait pathology for a single gait variable [8]. In patients with USCP, reported improvement of mean GVS across the studies’ samples ranged from 8.3 to 12.3, while the improvement in patients with BSCP ranged from 7.3 to 15.6. In USCP, improvement in GVS ranged from 83% to 100%, while in BSCP, improvement ranged from 81% to 93%.

Eight studies [16,19,20,23,29,30,32,33] reported Gait Profile Scores (GPS). The GPS measures the difference between patients and people with no gait pathology across nine kinematic variables [8]. All but two studies reported an improvement in GPS greater than the minimal clinically important difference (MCID) of 1.6° [41]. Pilloni et al. [19] reported a 1.5° deterioration in mean GPS in true equinus compared to 6.4° improvement in jump gait. Kim et al. [29] investigated endoscopic surgery, reporting an improvement in GPS of 1.4°. Excluding these studies, the improvement of mean GPS in patients with USCP ranged from 1.8° to 3.8°, while improvement in BSCP ranged from 2.2° to 6.6°. Post-operatively, improvement in GPS was noted in 70 to 100% of patients with USCP and 86% of patients with BSCP.

### 3.3. Stability of Correction

Twenty-one studies (Table 1) reported the presence of recurrent equinus, calcaneal gait, or crouch gait following calf lengthening surgery. Definitions varied and were based on either stance or swing phase, with different distances from the mean of normal used. In children with USCP, recurrent equinus ranged from 0% to 38%, and calcaneal gait from 4% to 30%. In children with BSCP, recurrent equinus ranged from zero to 35%, and calcaneal gait from 0% to 40%. Recurrent equinus ranged from 0% to 18% in studies with less than four years of follow up, and 16% to 38% in studies with more than four years of follow up. For calcaneus, studies with less than four years of follow up reported a rate of 0% to 30%, while studies with more than four years follow up reported a rate of 3% to 40%.

**Table 1:** Studies of calf lengthening surgery.

Study	CP Type/ No. of Subjects	Type of Surgery Eponym and Zone ^†^	Stability of Correction	Follow Up in Months: Mean (Range)	Outcome Measures	MINORS
Baddar 2002 [14]	34B	Isolated Zone 2	-	15 (9–23)	CE, 3DGA, EMG, gastroc-soleus length, SPK	8
Borton 2001 [15]	45U, 89B	Isolated Zone 2/3 (Baker/ZTAL/Hoke TAL)	Recurrent equinus: U = 38%, B = 16% Calcaneus: U = 4%, B = 40%	(60–120)	CE, 3DGA, PRS	11
Cimolin 2011 [37]	10U, 9B	Isolated Zone 2 (modified Vulpius)	-	13	3DGA, GDI	9
Davids 2011 [4]	33U, 20B	Isolated + SEMLS Zone 1/3 (Strayer/White)	-	27 (20–65)	CE, 3DGA, SMC, SPK	10
Dreher 2012 [9]	44B	SEMLS Zone 1 (Baumann) to 15–20° DF in KE/KF	Recurrent equinus—24% Calcaneus—9% early onset (<1 year), 11% late onset (>1 year)	103 (12–156)	CE, 3DGA, SPK, GGI	10
Engsberg 2005 [27]	32B	Isolated Zone 2/3 (Vulpius/White)	-	14	CE, 3DGA	8
Etnyre 1993 [28]	11U, 13B	SEMLS Zone 1/2/3 (Strayer/Vulpius/Baker/Z-/step/sliding)	Recurrent equinus—12.5% (2U, 1B)	8	CE, EMG, 3DGA	6
Firth 2013 [16]	44B	SEMLS Zone 1/3 (Strayer/modified Strayer/White slide) to 5° DF with KE	Recurrent equinus—35% Calcaneal gait—2.5% Revision calf surgery—13%	48	CE, 3DGA, GVS, GPS	9
Fujita 2020 [23]	10U	SEMLS Zone 2 (Baker) + FHL transfer	Recurrent equinus—0% Calcaneus—30%	35 (25–64)	CE, 3DGA, SPK, GVS, GPS	7
Galli 2009 [10]	12B	Isolated Zone 2 (modified Vulpius) to neutral	Recurrent equinus—0%	(3–62)	CE, 3DGA, SPK	7
Galli 2005 [17]	8U, 12B	Isolated Zone 2 (modified Vulpius) to neutral	-	12 (+/−2)	CE, 3DGA, SMC	7
Jahn 2009 [42]	Not reported	Isolated Zone 2/3 (Vulpius/TAL)	-	13 (8–20)	CE, 3DGA, muscle lengths	7
Kay 2004 [35]	23U, 32B	SEMLS Zone 1/3 (GR/TAL)	Under-correction—3–28% Over-correction—19–22%	19 (+/−10)	CE, 3DGA, SMC, PRS, SPK	7
Kim 2020 [29]	14B	SEMLS Zone 2 (Endoscopic/open modified Vulpius) to 10° DF in KE	Surgical complications—none Recurrent equinus—7% Overcorrection—0%	24	CE, 3DGA, GDI, GPS	13 ^‡^
Klausler 2017 [30]	12U, 8B	SEMLS Zone 3 (TATS + ZTAL) to 10° DF for U, 5–10° PF for B	Recurrent equinus—13% (3U) Overcorrection—0%	70	CE, 3DGA, GPS	8
Klotz 2016 [31]	18B	SEMLS Zone 1/3 (Baumann/Strayer/Z/Hoke)	-	13	CE, 3DGA	9
Klotz 2013 [43]	19B	SEMLS Zone 1/3 (Baumann/Strayer/Z/Hoke)	-	14	CE, 3DGA	7
Lofterod 2009 [12]	16U, 18B	SEMLS Zone 2/3 (Vulpius/ZTAL) to 10° DF in KE	Persistent drop foot—48% Undercorrection—15% Overcorrection—2.5%	(11–21)	CE, 3DGA, FMS, SPK	7
Lofterod 2008 [18]	6U, 9B	Isolated Zone 2/3 (Vulpius/ZTAL) to 10° DF in KE	Recurrent equinus—5% (at 3 years) Under-correction—20% Over-correction—5%	(13–55)	CE, 3DGA, SPK	7
Park 2006 [24]	16U	Single level Zone 2/3 (?/TAL)	-	15 (9–25)	CE, 3DGA, SPK	7
Patikas 2007 [39]	16U, 18B	SEMLS	-	30	CE, EMG, 3DGA, GGI	8
Pilloni 2019 [19]	18B	Isolated Zone 1 (bilateral modified Strayer)	Overcorrection—75% of true equinus	12	CE, 3DGA, GVS, GPS	8
Rajagopal 2020 [44]	Not reported	SEMLS	-	14	CE, 3DGA, ADI	14 ^‡^
Rose 1993 [36]	5U, 15B	SEMLS Zone 2 (modified Baker) to 10° DF in KE	Crouch gait—0%	13	CE, 3DGA, SPK	7
Rutz 2011 [32]	21U, 8B	SEMLS Zone 3 (TATS + ZTAL) to 10° DF for U, 5–10° PF for B	Surgical complications—none	14	CE, 3DGA, GPS, GDI, GGI	9
Rutz 2020 [45]	NA	-	-	-	Delphi consensus study	NA
Saraph 2000 [25]	22B	SEMLS Zone 1 (Baumann) to neutral	Recurrent equinus—0% Under-correction—5% Over-correction—0%	(25–48)	CE, 3DGA, SPK	5
Skaaret 2019 [33]	33U	SEMLS Zone 1/3 (TAL)	-	16 (11–27)	CE, 3DGA, SMC, SPK, GPS, GVS	8
Steinwender 2001 [26]	29B	SEMLS Zone 1 (Baumann)	-	47	CE, 3DGA, SPK	14 ^‡^
Svehlik 2012 [11]	18B	SEMLS Zone 1 (Baumann) to neutral in KE	Recurrent equinus—24% Overcorrection—10%	120	CE, 3DGA, GDI	8
Tinney 2015 [20]	12U, 14B	Single level: U SEMLS: B Zone 2 (Vulpius) to 10° DF in KE	Surgical complications—none Recurrent equinus: U = 17% B = 14% Calcaneus: U = 17% B = 7%	12	CE, 3DGA, GPS, GVS	9
Tylkowski 2009 [21]	13U, 14B	Isolated Zone 3 (Hoke) to neutral	Under-correction—0% Over-correction—0%	14	CE, 3DGA, SPK, oxygen cost	8
Vuillermin 2011 [34]	27B	Single level Zone 2/3 SEMLS Zone 1 (Strayer)	Decreased crouch gait in second 5 year period studied	180	CE, 3DGA	10
Wren 2004 [22]	3U, 9B	Zone 1: gastrocnemius recession	-	13	CE, 3DGA, muscle lengths	12 ^‡^

CP = cerebral palsy, U = unilateral spastic cerebral palsy, B = bilateral spastic cerebral palsy, SEMLS = single-event multi-level surgery, KE = knee extension, KF = knee flexion, GR = gastrocnemius recession, TAL = tendo-achilles lengthening, CE = clinical examination, 3DGA = three-dimensional gait analysis, SPK = swing phase kinematics, PRS = physician rating scale, GGI = Gillette Gait Index, GPS = gait profile score, GVS = gait variable score, GDI = gait deviation index, ADI = ankle deviation index, FMS = functional mobility score, ^†^ Three zone classification according to Firth 2013 [46], ^‡^ Comparative studies. Please see reference [1] for a more detailed description of SEMLS and the role of 3DGA.

**Table 2:** Surgical studies reporting gait scores out of 34 total studies.

	GPS/GVS	GDI	GGI	Unilateral/Bilateral
Cimolin 2011 [37]	-	Y	-	Pooled U, B
Dreher 2012 [9]	-	-	Y	B
Firth 2013 [16]	Y	-	-	B
Fujita 2020 [23]	Y	-	-	U
Kim 2020 [29]	Y	Y	-	B
Klausler 2017 [30]	Y	-	-	U, B
Patikas 2007 [39]	-	-	Y	U, B
Pilloni 2019 [19]	Y	-	-	B
Rutz 2011 [32]	Y	Y	Y	U, B
Skaaret 2019 [33]	Y	-	-	U
Svehlik 2012 [11]	-	Y	-	B
Tinney 2015 [20]	Y	-	-	U, B

GPS: Gait Profile Score, GVS: Gait Variable Score, GDI: Gait Deviation Index, GGI: Gillette Gait Index, U: unilateral spastic cerebral palsy, B: bilateral spastic cerebral palsy, Y: yes.

### 3.4. Study Quality

**Figure 4:**
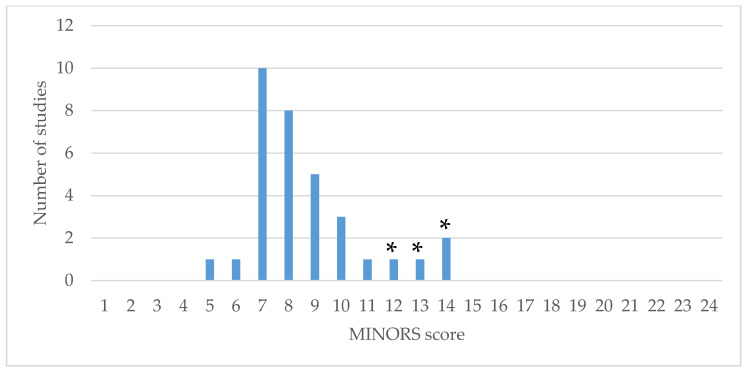
MINORS scores for 33 surgical cohort studies. * Comparative studies. Note: Cohort studies have a maximum score of 16. Comparative studies have a maximum score of 24.

Evaluation of the 33 cohort studies using MINORS showed marked variability in the quality of the studies (Figure 4). Scores for cohort studies ranged from six to 11 (maximum score 16), with no studies being blinded, none reporting any prospective calculation of study size and most studies being retrospective.

## 4. Discussion

Introduction of 3DGA has assisted pre-operative planning, detailed analysis of pre-operative gait pattern and objective assessment of outcomes following surgery for equinus gait, in children with CP. Correction of equinus is important to improve quality of life in patients, as well as prevent further musculoskeletal consequences such as midfoot break, which can result in pain, calluses, and brace intolerance making walking and wearing of supportive footwear difficult [47]. In 2020, Rutz et al. [45] reported in a Delphi study a process to achieve consensus between experienced surgeons on the identification of factors, including 3DGA that provide indications for GSL surgery. Pre-operative predictors for surgical outcomes are important for informed decisions to be made about interventions. Improvement in ankle kinematics by calf lengthening surgery during stance phase is well described in children with USCP and BSCP [9,10,11,14,24,25,26,27,33]. However, post-operative changes in swing phase are also important as insufficient foot clearance during swing can lead to tripping, and compensatory movements such as increased knee or hip flexion [18]. A drop foot in swing may require the use of an AFO and is perceived as a “limp” or abnormal gait pattern by patients, peers and parents/carers [1].

All studies in this review reported an improvement in equinus gait during stance phase and the improvements were clinically and statistically important in the majority of studies [10,11,14]. All studies in this review found improved ankle dorsiflexion in swing, in some patients. Lofterod et al. reported that 47.5% of their patients still exhibited drop foot during swing after calf lengthening [12]. This study suggested a relationship between increased pre-operative maximum plantarflexion in initial swing and post-operative drop foot. It also suggested that a pre-operative Selective Motor Control (SMC) score of four using the Boyd and Graham score [48] is equivalent to normal swing phase post-operatively. However, this was based on only four limbs, so care should be taken with making generalisations from these limited numbers.

Davids et al. [4] failed to identify any predictors for active ankle dorsiflexor function in swing, defined by a positive slope of the sagittal ankle kinematic graph during swing phase. Presence of active ankle dorsiflexion in swing increased post-operatively from 79% to 96% of patients, with 19% of patients improving from a grading of “absent” to “present”, and 2% changing from “present” to “absent”. Davids et al. also reported 38% of patients improving in SMC, with 40% of patients graded as having normal SMC pre-operatively and 53% of patients having normal SMC post-operatively. Dynamic electromyography (EMG) showed no clear improvement in gastrocnemius and tibialis anterior co-activation during swing, with 11% of patients having abnormal co-activation pre-operatively, and a different 11% having abnormal co-activation post-operatively. The absence of change in EMG activity post-operatively despite improvement in swing phase kinematics is supported by other studies [14,39]. While drop foot in swing phase was not an outcome measured in the study, these findings suggest that some patients are able to exhibit active ankle dorsiflexor function without normal SMC or normal phasic activation of the gastrocnemius and tibialis anterior. Further investigation of muscle activation patterns is required as excessive co-contraction of agonist and antagonist muscles, specifically the dorsiflexor and plantarflexor muscles in the case of equinus and drop foot, play a role in the mechanism of spastic paresis [5]. The role of peroneus longus (PL) in drop foot could be considered as premature onset of PL activity in swing phase in conjunction with premature gastrocnemius activity has been implicated in equinovalgus deformity [3].

Kay et al. [35] also found no significant improvement in dorsiflexor control, despite improvement in swing phase dorsiflexion. This casts further doubt on the role of SMC in predicting swing phase kinematics.

In 2020, Rajagopal et al. [44] reported greater improvement of ankle kinematics after GSL in patients with short gastrocnemius lengths in gait pre-operatively, compared to patients without a short gastrocnemius. However, the zone of GSL used for each patient was not reported and the patient sample was not classified by CP type. Additionally, the use of the ankle deviation index (ADI) as the outcome measure did not allow information about swing phase dorsiflexion to be extracted. Hence, further investigation is required for the use of gastrocnemius muscle length as a pre-operative predictor of GSL outcomes.

It is well accepted that surgical outcomes differ between CP topographical types, USCP and BSCP [45]. However, none of the aforementioned studies distinguished patients according to topographical distribution, USCP or BSCP. Hence, further studies investigating swing phase kinematics should consider analysis of outcomes based on CP subtype and movement disorder.

Gait scores provide an objective measure of the deviation of a patient’s gait from that of a typically developing child, as well as averaging kinematics at multiple joints. This is important as SEMLS is the most widely used surgical intervention in children with BSCP [1]. The gait outcomes of SEMLS are the result of corrections of deformities in multiple locations and anatomic planes, not just GSL (Figure 1). Global gait scores reported by studies [32,39] suggest that patients with BSCP have a more abnormal global gait pattern than patients with USCP pre-operatively, allowing more room for improvement [1]. This is supported by greater post-operative improvement in gait found in these studies in patients with BSCP compared to those with USCP.

Under-correction after calf lengthening surgery can lead to recurrent equinus, while over-correction can lead to calcaneus or crouch gait. Shore et al. [49] highlighted that cerebral palsy type, surgical technique, and follow-up period can influence the rate of recurrent equinus and calcaneus. This review supports the finding that studies with longer follow-up periods had higher rates of recurrence or calcaneus, emphasising the need for longer follow-up periods, preferably to skeletal maturity and beyond, to determine the stability of correction more accurately. However, this review did not find any obvious difference between the rates of recurrent equinus and calcaneal gait when comparing USCP and BSCP. This may be due to the range of sample sizes, follow-up periods, surgeries and definitions used.

Type of surgery can impact the rates of calcaneus and recurrent equinus after surgery. Results from Fujita et al. [23] suggest that anterior transfer of flexor hallucis longus with gastrocsoleus lengthening can increase the rate of post-operative calcaneus. Additionally, Borton et al. [15] reported post-operative calcaneal gait in 40% of patients with BSCP. This is much higher than other studies of similar follow-up period that reported rates of 10% to 11% [9,11]. The higher rate of calcaneus reported by Borton et al. may be due to the use of isolated zone 2 and 3 calf lengthening surgeries and inclusion of patients as young as two years old (Figure 2). Zone 3 surgeries are now considered contraindicated for patients with BSCP, and there is consensus that the preferred age for calf lengthening surgery is between six and ten years old [45]. Multiple features from the Delphi study have been suggested to optimise the indications for GSL, including age at surgery, CP subtype, physical examination, and kinematic features [45]. These criteria remain to be tested in prospective studies and clinical trials.

In 2011, Rutz et al. described a novel technique utilising concurrent tibialis anterior tendon shortening (TATS) with calf lengthening to improve dorsiflexor function [32]. The improvements in GDI and GVS fell within the range found by other studies, with the exception of a greater improvement in GPS in patients with BSCP [11,19,20,23,29,37]. This makes interpretation difficult as to whether TATS provides additional benefit to ankle kinematics, when added to GSL. However, using TATS, Klausler et al. reported 0% overcorrection at a mean six-year follow-up period, with 25% of patients with USCP and 0% of patients with BSCP having recurrent equinus [30]. This is lower than other reported recurrent equinus rates of 38% in USCP and 16–35% in BSCP at similar follow-up periods [9,11,15,16]. Overcorrection or calcaneal gait at long-term follow-up has been reported as 4% in children with USCP and 3–11% in children with BSCP [9,11,15,16]. However, it should be noted that Klausler et al. did not provide a definition for recurrent equinus, overcorrection or calcaneal gait, which have been variably defined across studies [15,28,35].

Limitations of this review include a single author screening studies for inclusion, extracting study characteristics and outcomes, and performing qualitative synthesis. This was addressed by using a systematic approach to the inclusion of articles (Figure 3). Additionally, the studies included in this review were of generally poor methodological quality (Figure 4). Study samples and interventions were heterogenous and ranges reported in this review include outcomes of studies with variable sample characteristics and incomplete documentation of key variables.

Improvement in study design is critical for future studies. Gait patterns involve multiple joints, and interventions are tailored to the individual, making rigorous study designs challenging to conduct. However, while cohort studies remain the dominant design for investigating surgical interventions, more rigorous evidence could be provided by the use of prospective studies and longer follow-up periods. Comparison of studies could be improved by agreed, universal definitions for equinus, recurrent equinus, calcaneus, and crouch gait based on physical examination and 3DGA parameters. Further breakdown of outcome measures based on topographic distribution of CP, Rodda and Graham equinus classifications [50] and calf lengthening zone could provide useful information in predicting outcomes. Finally, predictors of post-operative drop foot in swing, and recurrent equinus or calcaneus requires further exploration.

## 5. Conclusions

Various methods of calf lengthening surgeries are available to treatment fixed equinus deformities in children with cerebral palsy. Studies using 3DGA provide objective outcome measures but vary greatly in sample characteristics, with many studies pooling patients and interventions without considering the impact of features such as topographic distribution and type of equinus. Further prospective studies and randomised control trials to determine pre-operative predictors for swing phase kinematics could consider CP type, equinus type, type of surgery, active ankle dorsiflexor function, gastrocnemius muscle length, muscle activation patterns, and SMC.

## Data Availability

The data presented in this study are available in the Supplementary Material.

## Supplementary Materials

The following are available online at https://www.mdpi.com/1010-660X/57/2/98/s1.
