# Supplementary material for: Three-Dimensional Gait Analysis in Children Undergoing Gastrocsoleus Lengthening for Equinus Secondary to Cerebral Palsy
Source: Medicina (Kaunas). 2021 Jan 22;57(2):98. doi: 10.3390/medicina57020098 (PMC7911110; doi:10.3390/medicina57020098)
Supplement: Supplementary file 1 [file medicina-57-00098-s001.pdf]

Supplementary Material

Search strategies

Database: Ovid MEDLINE(R) and Epub Ahead of Print, In-Process & Other Non-Indexed Citations and Daily <1946 to August 25, 2020>

1 *Cerebral Palsy/ (16,828)

2 (Cerebral-pals* or dystonic or mixed-movement-disorder* or spastic-diplegi* or (little* adj disease) or brain-palsy or brain-paralysis or central-paralysis or cerebral-paralysis or cerebral-paresis or encephalopathia-infantilis).tw,kf. (27,896)

3 1 or 2 (29,655)

4 (Equinus or calf-muscle-spasticit* or contracture-of-triceps-surae or drop-foot).tw,kf. (2,026)

5 Equinus Deformity/su [Surgery] (210)

6 4 or 5 (2,086)

7 exp Orthopedic Procedures/ (307,109)

8 (surger* or surgical* or operat*).tw,kf. (2,637,610)

9 su.fs. (1,987,450)

10 (Conservative-treatment or conservative-therap* or ankle-foot-orthos#s or AFO or electrical-stimulation).tw,kf. (84,878)

11 (Gastrocnemius-soleus-intramuscular-aponeurotic-recession or tendon-lengthening or Baker-procedure or Baumann-procedure or calf-muscle-lengthening or gastrosoleus-aponeurotic-lengthening or tendo-achilles-lengthening or gastrocnemius-recession).tw,kf. (664)

12 7 or 8 or 9 or 10 or 11 (3,680,086)

13 (Gait-analys* or 3DGA or kinematic* or kinetic* or motion-capture or gait-data or gait-profile-score or movement-analysis or motion-measurement-system or electromyograph* or gait-parameter*).tw,kf. (464,070)

14 (Selective-motor-control or coactivation or co-activation or muscle-strength or muscle-activation).tw,kf. (32,620)

15 exp "Range of Motion, Articular"/ (51,707)

16 exp Gait/ or Gait Disorders, Neurologic/ or Contracture/ (41,705)

17 exp Biomechanical Phenomena/ (130,872)

18 Motor Skills/ (24,330)

19 exp Motor Activity/ (291,323)

20 exp treatment outcome/ (1,058,127)

21 Muscle strength/ or muscle contraction/ (114,029)

22 13 or 14 or 15 or 16 or 17 or 18 or 19 or 20 or 21 (2,029,692)

23 (newborn* or new-born* or baby or babies or neonat* or neo-nat* or infan* or toddler* or pre-schooler* or preschooler* or kinder or kinders or kindergarten* or kinder-aged or boy or boys or girl or girls or child or children or childhood or pediatric* or paediatric* or school-age* or schoolage* or schoolchild* or schoolgirl* or schoolboy* or adolescen* or youth or youths or teen or teens or teenage*).tw,kf. (2,384,201)

24 3 and 6 and 12 and 22 and 23 (124)

25 limit 24 to (english language and yr="1990 -Current") (103)

Database: Embase Classic+Embase <1947 to 2020 August 25>

1 cerebral palsy/ (41,101)

2 (Cerebral-pals* or dystonic or mixed-movement-disorder* or spastic-diplegi* or (little* adj disease) or brain-palsy or brain-paralysis or central-paralysis or cerebral-paralysis or cerebral-paresis or encephalopathia-infantilis).tw,kw,dq. (43,152)

3 1 or 2 (52,013)

4 (Equinus or calf-muscle-spasticit* or contracture-of-triceps-surae or drop-foot).tw,kw,dq. (3,070)

5 Equinus Deformity/su (271)

6 4 or 5 (3,146)

7 exp orthopedic surgery/ (522,219)

8 (surger* or surgical* or operat*).tw,kw,dq. (3,710,020)

9 su.fs. (2,177,641)

10 (Conservative-treatment or conservative-therap* or ankle-foot-orthos#s or AFO or electrical-stimulation).tw,kw,dq. (121,352)

11 (Gastrocnemius-soleus-intramuscular-aponeurotic-recession or tendon-lengthening or Baker-procedure or Baumann-procedure or calf-muscle-lengthening or gastrosoleus-

aponeurotic-lengthening or tendo-achilles-lengthening or gastrocnemius-recession).tw,kw,dq. (959)

12 7 or 8 or 9 or 10 or 11 (5,014,627)

13 (Gait-analys* or 3DGA or kinematic* or kinetic* or motion-capture or gait-data or gait-profile-score or movement-analysis or motion-measurement-system or electromyograph* or gait-parameter*).tw,kw,dq. (554,597)

14 (Selective-motor-control or coactivation or co-activation or muscle-strength or muscle-activation).tw,kw,dq. (45,346)

15 exp "joint characteristics and functions"/ (96,607)

16 gait/ or exp neurologic gait disorder/ or exp contracture/ (91,594)

17 biomechanics/ (111,823)

18 motor performance/ (78,498)

19 exp motor activity/ (581,343)

20 exp treatment outcome/ (1,679,336)

21 muscle strength/ or muscle contraction/ (136,688)

22 13 or 14 or 15 or 16 or 17 or 18 or 19 or 20 or 21 (3,117,001)

23 (newborn* or new-born* or baby or babies or neonat* or neo-nat* or infan* or toddler* or pre-schooler* or preschooler* or kinder or kinders or kindergarten* or kinder-aged or boy or boys or girl or girls or child or children or childhood or pediatric* or paediatric* or school-age* or schoolage* or schoolchild* or schoolgirl* or schoolboy* or adolescen* or youth or youths or teen or teens or teenage*).tw,kw,dq. (3,215,377)

24 3 and 6 and 12 and 22 and 23 (205)

25 limit 24 to (english language and yr="1990 -Current") (176)

Database: PubMed

#1 Title/Abstract

“Cerebral-pals*” OR “dystonic” OR “mixed-movement-disorder*” OR “spastic-diplegi*” OR “little-disease” OR “little’s-disease” OR “littles-disease” OR “brain-palsy” OR “brain-paralysis” OR “central-paralysis” OR “cerebral-paralysis” OR “cerebral-paresis” OR “encephalopathia-infantilis”

#2 Title/Abstract

“Equinus” OR “calf-muscle-spasticit*” OR “contracture-of-triceps-surae” OR “drop-foot”

#3 Title/Abstract

“surger*” OR “surgical*” OR “operat*” OR “procedure*” OR “conservative-treatment” OR “conservative-therap*” OR “ankle-foot-orthosis” OR “ankle-foot-orthoses” OR “AFO” OR “electrical-stimulation” OR “Gastrocnemius-soleus-intramuscular-aponeurotic-recession” OR “tendon-lengthening” OR “Baker-procedure” OR “Baumann-procedure” OR “calf-muscle-lengthening” OR “gastrosoleus-aponeurotic-lengthening” OR “tendo-achilles-lengthening” OR “gastrocnemius-recession”

#4 Title/Abstract

“Gait” OR “3DGA” OR “kinematic*” OR “kinetic*” OR “motion-capture” OR “movement-analysis” OR “motion-measurement-system” OR “electromyograph*” OR “selective-motor-control” OR “coactivation” OR “co-activation” OR “muscle-strength” OR “muscle-activation” OR “Range-of-Motion” OR “Contracture” OR “biomechanic*” OR “Motor-skill*” OR “Motor-Activit*” OR “outcome*” OR “Muscle-strength” OR “muscle-contraction” OR “joint*” OR “motor-performance”

#5 Title/Abstract

“newborn*” OR “new-born*” OR “baby” OR “babies” OR “neonat*” OR “neo-nat*” OR “infan*” OR “toddler*” OR “pre-schooler*” OR “preschooler*” OR “kinder” OR “kinders” OR “kindergarten*” OR “kinder-aged” OR “boy” OR “boys” OR “girl” OR “girls” OR “child” OR “children” OR “childhood” OR “pediatric*” OR “paediatric*” OR “school-age*” OR “schoolage*” OR “schoolchild*” OR “schoolgirl*” OR “schoolboy*” OR “adolescen*” OR “youth” OR “youths” OR “teen” OR “teens” OR “teenage*”

#6 All fields

NOTNLM OR publisher[sb] OR inprocess[sb] OR pubmednotmedline[sb] OR indatareview[sb] OR pubstatusaheadofprint

#7 #1 AND #2 AND #3 AND #4 AND #5 AND #6

#8 Title/Abstract

limit to english language

Results = 37
